# Comparative analysis of language models in addressing syphilis-related queries

**DOI:** 10.4317/medoral.27092

**Published:** 2025-05-27

**Authors:** Luiz Miguel Ferreira, João Pedro Santos Nascimento, Lucas Lacerda de Souza, Fabrício Tinôco Alvim de Souza, Letícia Drumond de Abreu Guimarães, Márcio Ajudarte Lopes, Pablo Agustin Vargas, Hercílio Martelli-Júnior

**Affiliations:** 1 Department of Oral Diagnosis, Piracicaba Dental School, University of Campinas, Piracicaba/Brazil; 2 Department of Dental Clinic, Dental School, Federal University of Juiz de Fora, Juiz de Fora/Brazil; 3 Health Science/Primary Care Postgraduate Program, State University of Montes Claros (Unimontes), Montes Claros/Brazil

## Abstract

**Background:**

Syphilis, caused by *Treponema pallidum*, is a significant global health concern with potentially severe complications if untreated. Advances in artificial intelligence (AI), particularly large language models (LLMs), offer opportunities to enhance medical diagnosis and public health education. This study aims to assess LLMs' ability to provide readable, accurate, and comprehensive syphilis information by comparing it with WHO datasheets and validating through specialist evaluation for clinical relevance.

**Material and Methods:**

Ten AI-based LLMs were evaluated. Ten questions addressing symptoms, transmission, diagnosis, treatment, and prevention were crafted by researchers. Responses from the LLMs were compared to World Health Organization (WHO) syphilis fact sheets, and a panel of specialists assessed the accuracy, clinical relevance, and readability of the AI-generated information.

**Results:**

Among the evaluated LLMs, ChatGPT 4.0 and Claude demonstrated the highest accuracy, scoring 92% and 89% alignment with WHO standards, respectively. Perplexity and Llama3 performed less reliably, with scores between 60-70%, especially in areas like tertiary syphilis and neurosyphilis. Specialists identified common errors, such as outdated treatment protocols and incorrect descriptions of transmission pathways. Expert reviews further revealed that while LLMs provided adequate information on early syphilis symptoms, they struggled with complex clinical nuances. The specialists' evaluation showed that only 60% of the AI-generated content was deemed clinically reliable without further edits, with ChatGPT 4.0 rated highest by experts in terms of readability and clinical accuracy.

**Conclusions:**

LLMs hold promise for disseminating syphilis information, but human oversight is crucial. AI models need refinement to improve their accuracy, especially in complex medical scenarios.

** Key words:**Artificial intelligence, large language models, pathology, syphilis.

## Introduction

Syphilis is a sexually transmitted infection caused by the bacterium *Treponema pallidum* (1,2). It has been a significant public health issue worldwide, being responsible for approximately 70,000 deaths globally, but this number might be an underestimate due to reporting inconsistencies (3,4). The disease unfolds in stages, beginning with primary and secondary phases that are highly infectious. If not treated, syphilis can progress to latent and tertiary stages, leading to severe complications (5).

Syphilis begins with primary stage, marked by a painless but highly infectious chancre, often unnoticed (6). The secondary stage includes generalized symptoms like a widespread rash, mild fever, fatigue, and swollen lymph nodes (2). If untreated, symptoms fade, leading to a latent phase where the bacterium remains dormant (3). Without treatment, the disease can develop to tertiary stage, causing severe cardiovascular and neurological damage (7). Neurosyphilis, which can occur at any stage, is particularly severe and includes symptoms like headaches, motor coordination difficulties, paralysis, or sensory deficits (8). Furthermore, the disease poses significant risks during pregnancy, potentially leading to congenital syphilis which can cause miscarriage, stillbirth, or severe developmental issues in the child (9,10).

The development of artificial intelligence (AI) technologies has revolutionized the approach to diagnosing infectious diseases, including syphilis. By integrating advanced AI algorithms with medical imaging and data analysis, these technologies can rapidly identify patterns and anomalies that may elude human observers, thereby enhancing diagnostic accuracy and speed (11-13). Particularly, AI systems are trained on diverse datasets to recognize the various stages of syphilis from clinical images and patient data, facilitating early and accurate diagnoses which are crucial for effective treatment (14).

In this context, large language models (LLMs) play a pivotal role by disseminating complex medical information in an accessible manner. They serve both the general public and specialists by providing up-to-date medical knowledge, guidelines, and research outcomes (15). For the public, LLMs can offer explanations about symptoms and preventive measures, while healthcare professionals can utilize these models for deeper insights into disease management strategies and the latest advancements in the field (12). However, the complex nature of AI analysis is difficult to understand because its learning requires a very broad collection of data, and the answers provided by the machine may be incorrect or biased (16,17). Thus, there are questions about the reliability of AI diagnoses, as well as the underlying foundations for assessing the accuracy of this technology (16).

Given the growing use of LLMs by the general population and the importance of early diagnosis and treatment of syphilis, including the fact that patients often seek alternative diagnostic methods due to shame or fear, as there is a social stigma surrounding sexually transmitted diseases affecting the genital organs (18), the objective of this study is to evaluate the readability, accuracy, and comprehensiveness of syphilis information provided by LLMs, comparing it with information WHO datasheet, and incorporating specialist evaluations to ensure clinical accuracy and relevance.

## Material and Methods

This cross-sectional study was approved by the Research Ethics Committee under registration number 6.786.028.

- Study Design

After carefully preparing a series of prompts, questions were posed to the LLMs to evaluate their ability to generate accurate and relevant information about syphilis. The responses produced by the LLMs were then meticulously analyzed and compared by specialists in the field. These experts compared the AI-generated answers to the standard reference answers, which were developed based on the WHO Fact Sheets on Syphilis (https://www.who.int/news-room/fact-sheets/detail/syphilis). These WHO fact sheets, which are freely accessible on the internet, serve as a globally recognized source of accurate and up-to-date information on the disease.

- Questions Elaboration

Given the complexity and importance of accurate information on syphilis, a set of ten carefully crafted questions was developed by a team of three experienced researchers (as detailed in Table 1). These questions were designed to cover various aspects of syphilis, including its symptoms, transmission, diagnosis, treatment, and prevention, ensuring a comprehensive assessment of the AI models’ capabilities. To mitigate any potential bias and to maintain the integrity of the evaluation process, the researchers deliberately avoided using AI tools or models in the creation of these questions. This decision was crucial to ensure that the questions were neutral and did not inadvertently favor the strengths or weaknesses of any particular AI model.

- Selection of AI Platforms

The platforms selected for this study - ChatGPT 3.5 (https://chatgpt.com/), ChatGPT 4.0 (https://chatgpt.com/), ScholarGPT (https://chatgpt.com/g/g-L2HknCZTC-scholar-ai), Gemini (https://gemini.google.com/), Llama3 (https://llama.meta.com/llama3/), BingChat (https://www.bing.com/chat), Perplexity (https://www.perplexity.ai/), Pop-AI (https://www.popai.pro/), Claude (https://claude.ai/), and ReKa Core (https://www.reka.ai/) - were specifically chosen due to their widespread popularity as AI-driven chatbots and their significant usage by the general public for generating responses to a wide range of queries. These platforms represent some of the most advanced Large LLMs available today, each with its unique approach to processing and generating human-like responses based on vast datasets and advanced algorithms. Their selection was driven by their reputation for being user-friendly, widely accessible, and frequently utilized by millions of users globally for information retrieval, problem-solving, and conversational AI applications.

- Selection of Evaluators

To ensure a rigorous and standardized evaluation of the responses generated by the AI platforms, three experts holding PhDs in Oral Pathology and Oral Medicine were carefully selected to participate in the study. These professionals were chosen based on their extensive expertise and deep understanding of syphilis, particularly as it relates to oral manifestations and overall pathology.

- Prompting

**Figure 1:**
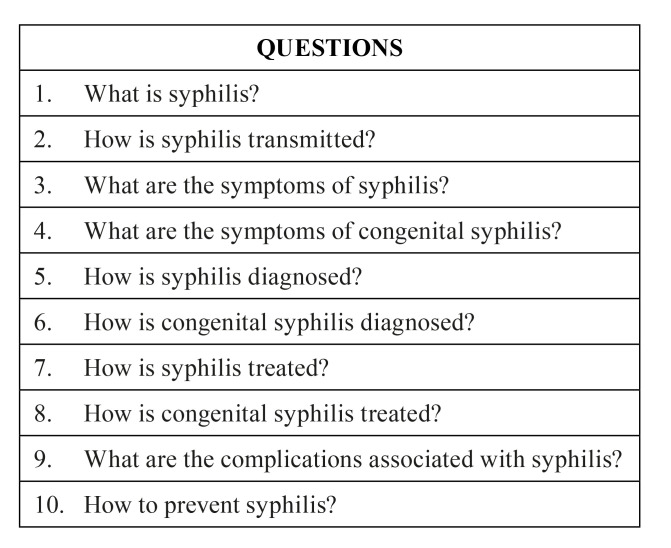
Workflow for the comparative analysis of language models in addressing syphilis-related queries.

English was the only language used on the platforms to ensure consistency in the responses. Each of the ten questions was submitted to the AI in a new chat window to prevent memory retention bias. To set the context, three prompts were sent to the AI before each main question: 1) “Take on the role of a healthcare professional to answer the questions”: This prompt guided the AI to provide responses with the expertise expected from a healthcare provider.; 2) “We will provide questions about syphilis and we want your help”: This prompt clarified the topic, ensuring the AI understood that the focus was on syphilis-related questions; 3) “Answer the questions concisely”: This prompt encouraged the AI to deliver clear and to-the-point answers. These steps were taken to create a consistent and unbiased testing environment, ensuring that each AI model was evaluated fairly (Fig. 1).

- Evaluation of responses

The evaluators were blinded, and the responses were randomized to prevent bias and avoid potential identification of patterns specific to each LLM. The first question on the rating form asked the evaluators to choose between two responses: one provided by an LLM and the other by the WHO. Following this, the evaluators were instructed to first evaluate the completeness of the response. A three-point scale was used to measure completeness as follows: 1) Incomplete: Addresses some aspects of the question but lacks significant parts or is incomplete. 2) Adequate: Addresses all aspects of the question and provides the minimum amount of information necessary to be considered complete. 3) Comprehensive: Addresses all aspects of the question and provides additional information or context beyond what is expected (19).

For accuracy, the following Likert scale (20) was employed: 1) Completely incorrect. 2) More incorrect than correct. 3) Approximately equal parts are correct and incorrect. 4) More correct than incorrect. 5) Completely correct. This structured approach ensured that both the completeness and accuracy of the AI-generated responses were thoroughly and fairly evaluated. In addition, after the evaluation of two specialists, a third specialist was consulted in case of divergence.

- Statistical Analysis

The responses provided by WHO and the LLMs for each question were systematically evaluated and compared based on Word Count (WC) and readability, utilizing the Flesch-Kincaid Grade Level (FKGL) and Flesch Reading Ease Score (FRES). To assess the similarity and precision of the responses, several statistical analyses were conducted, including Cosine similarity to evaluate thematic similarity, Levenshtein distance to measure textual similarity by calculating the minimum single-character changes needed to transform one text into another, and the Jaccard similarity coefficient to analyze the overlap of keyword sets, indicating conceptual similarity. Additionally, Cohen’s kappa coefficient was applied to assess the consistency of results across the questionnaires. All statistical analyses were performed using Python software (version 3.10.2; Python Software Foundation), ensuring a robust and standardized approach to the evaluation.

## Results

- Word count, Flesch Reading Ease Scores and Flesh-Kincaid Grade Level 

The analysis of WC shows that ReKa Core generated the longest responses with an average of 202.9 words, followed by Perplexity with 133 words and Bing Chat with 105.2 words. ChatGPT 4.0 and ChatGPT 3.5 produced 95.2 and 91.3 words on average, respectively. On the lower end, WHO generated 78.8 words, Claude produced 72.6 words, and POP-AI averaged 67.3 words. ScholarGPT generated 59.4 words, while Gemini and Llama 3 produced the shortest responses, with averages of 46 and 42.7 words, respectively.

The FRES indicates that ReKa Core and Bing Chat produced the most readable text with scores of 31.42 and 30.79, respectively, while Llama 3 generated the most complex text with a score of 13.51, followed by Perplexity at 17.9. Models like ScholarGPT (27.43), POP-AI (27.08), and WHO (26.95) produced moderately complex text, whereas ChatGPT 3.5 (21.41), ChatGPT 4.0 (20.25), and Gemini (20.45) fell in between.

**Figure 2:**
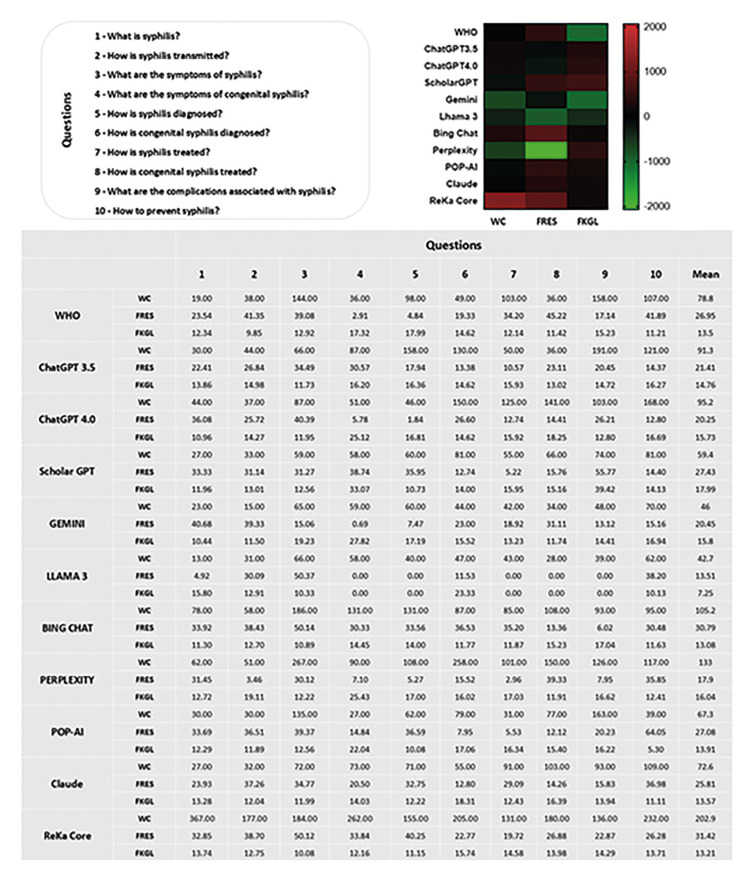
Results for WC, FRES and FKGL evidencing the main results and the average count for each of the questions. The heatmap evidenced that Perplexity produces higher WC but more complex text (higher FKGL, lower FRES). ChatGPT 4.0 and Gemini offer a balance with moderate WC and easier readability (higher FRES, lower FKGL). WHO, POP-AI, and ReKa Core generate shorter, more complex text (low FRES, high FKGL).

The FKGL analysis shows that ScholarGPT produced the most advanced text, with a grade level of 17.99, followed by Perplexity (16.04), Gemini (15.8), and ChatGPT 4.0 (15.73), indicating text suiTable for college-level readers. ChatGPT 3.5 generated text at a slightly lower level (14.76), while WHO (13.5), POP-AI (13.91), Claude (13.57), and ReKa Core (13.21) produced text at high school or early college reading levels. Bing Chat was the most accessible at a high school level (13.08), and Llama 3 generated the simplest text, suiTable for middle school readers, with a grade level of 7.25 (Fig. 2).

- Similarity Analysis

ChatGPT 3.5 achieved a Cosine similarity score of 0.666, with a Levenshtein distance of 505 and a Jaccard similarity coefficient of 0.496. ChatGPT 4.0 showed slight improvements, scoring 0.675 in Cosine similarity, 550 in Levenshtein distance, and 0.505 in Jaccard similarity. Scholar GPT followed with a Cosine similarity of 0.657, a lower Levenshtein distance of 428, and a Jaccard score of 0.488. Gemini recorded a Cosine similarity of 0.646, a Levenshtein distance of 392, and a Jaccard score of 0.476. Llama 3 had a Cosine similarity of 0.638, a Levenshtein distance of 398, and a Jaccard coefficient of 0.464.

Among the models, Bing Chat achieved the highest Cosine similarity at 0.683, though its Levenshtein distance was 535, with a Jaccard similarity of 0.516. Perplexity matched ChatGPT 3.5 in Cosine similarity at 0.666 but had a much higher Levenshtein distance of 686, with a Jaccard similarity of 0.488. POP-AI had a Cosine similarity of 0.645, a Levenshtein distance of 433, and a Jaccard coefficient of 0.472. Claude performed well with a Cosine similarity of 0.674, a Levenshtein distance of 438, and a Jaccard score of 0.506. Finally, ReKa Core had a Cosine similarity of 0.654, but its Levenshtein distance was the highest at 1073, with a Jaccard coefficient of 0.464.

**Figure 3:**
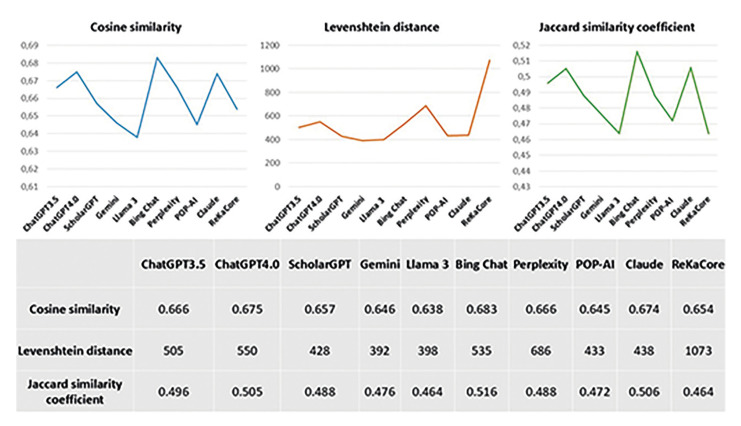
Summary of Cosine similarity, Levenshtein distance and Jaccard similarity coefficient.

Bing Chat, ChatGPT 4.0, and Claude seem to offer the best overall balance between semantic coherence and lexical similarity, while ReKa Core tends to diverge more significantly in lexical structure. Gemini and Scholar GPT may be preferable when lexical similarity is a higher priority (Fig. 3).

- Specialists Analysis

Cohen’s kappa coefficient was employed to assess the consistency between the evaluators across the questionnaires. The test showed the relation between evaluators one and two about the first question was substantial (κ = 0.680), about question two was slight (κ = 0.293), and in question three there was almost no agreement (κ = 0.060). Regarding question four, the agreement between the first and second evaluators was slight (κ = 0.154) and in question five was regular (κ = 0.247).

The evaluation of the models revealed that ReKa Core emerged as the most appropriate, achieving a rating of 9 out of 10 possible instances. Following closely were ChatGPT 3.5, ChatGPT 4.0, and Perplexity, each receiving a score of 8, while Bing Chat garnered a moderate score of 6. Claude and Pop-AI performed similarly, with both models rated appropriate in 5 instances. In contrast, GEMINI was deemed the least appropriate, with only 1 instance, and Llama 3.0 received a slightly higher but still lower-end score of 5. Regarding adherence to WHO guidelines, GEMINI was rated the highest, with 9 appropriate responses, underscoring its alignment with the guidelines, while ReKa Core received the lowest score of 1. ChatGPT 3.5, Llama 3.0, and Claude demonstrated moderate alignment, each scoring 5, while ChatGPT 4.0, Pop-AI, and Bing Chat showed some variation in adherence, scoring between 3 and 4 instances.

When assessing the appropriateness of the answers generated by the models, ScholarGPT and Llama 3.0 excelled in providing adequate responses, each recording 8 instances. ReKa Core stood out for delivering the highest number of comprehensive answers (5), while ChatGPT 4.0 also demonstrated competence in this area, contributing 3 comprehensive responses. Both Bing Chat and Perplexity were consistently adequate, with 7 responses rated as such. Conversely, GEMINI exhibited the highest number of incomplete answers, recording 4, followed by ChatGPT 3.5 with 2. However, ChatGPT 4.0, ScholarGPT, and Perplexity displayed robustness, each avoiding incomplete responses entirely. The instances of no agreement were minimal, with ChatGPT 3.5, Claude, and ReKa Core recording a few instances of disagreement.

In terms of responses based on WHO guidelines, the distribution was relatively balanced across the models. Perplexity provided the highest number of adequate answers, with 8, while Claude and ScholarGPT followed closely, contributing 7 adequate answers each. Other models, including ChatGPT 3.5, ChatGPT 4.0, Llama 3.0, GEMINI, and Bing Chat, produced 5 adequate answers, reflecting a moderate level of appropriateness. The incidence of incomplete answers was uniformly spread across most models, with ChatGPT 3.5, ChatGPT 4.0, ScholarGPT, GEMINI, Llama 3.0, and Bing Chat each contributing 3 incomplete responses. ReKa Core, however, recorded 4 incomplete answers. No model was rated as providing comprehensive WHO responses. Instances of no agreement were infrequent, with ChatGPT 3.5, ChatGPT 4.0, and GEMINI recording a few cases of disagreement.

The correctness evaluation revealed that ReKa Core and Perplexity were both rated as completely correct in 10 instances each, demonstrating a high level of accuracy. ChatGPT 4.0 and Bing Chat closely followed, each scoring 9 completely correct answers. Claude also performed exceptionally well, with 10 completely correct responses. ChatGPT 3.5, while solid, recorded 6 correct responses. On the other hand, ScholarGPT displayed some limitations, contributing only 2 completely correct answers, suggesting areas for potential improvement. Despite these disparities, ScholarGPT and GEMINI were noTable for providing more correct than incorrect answers, each recording 7 responses in this category. ChatGPT 3.5 and Llama 3.0 also exhibited moderate performance, each delivering 3 responses that were more correct than incorrect. Importantly, none of the models were found to be completely incorrect or more incorrect than correct, underscoring the general reliability of the models across all categories. Only ChatGPT 3.5 and ScholarGPT displayed minor discrepancies, with 1 instance each where no agreement was recorded.

The evaluation of WHO answers mirrored the trends observed in the LLM responses. Claude and ReKa Core stood out, delivering the highest number of completely correct answers, with 9 and 8, respectively. ChatGPT 3.5, ChatGPT 4.0, and ScholarGPT also performed competently, producing 5, 7, and 6 correct answers, respectively. Perplexity and Bing Chat maintained a strong performance, each offering 7 correct responses. Across all models, no instances of completely incorrect or more incorrect than correct answers were identified. Furthermore, there were no cases where the responses were approximately equal in correctness and incorrectness, emphasizing the robustness and reliability of the models in terms of correctness (Table 2).

## Discussion

AI has made significant progress in healthcare, particularly in diagnosing human diseases (21). LLMs, such as ChatGPT, can assist by processing large volumes of medical data and providing valuable insights to support clinical decision-making (12,15,21). The collaboration between AI and LLMs holds the potential to enhance diagnostic accuracy and improve the management of complex diseases, ultimately contributing to better healthcare outcomes (21). This is the first study to assess the accuracy, comprehensibility, and completeness of syphilis-related survey responses provided by ten widely used LLMs, both by the general public and researchers. It also differs from other studies by systematically comparing the quality of information provided by LLMs with WHO and expert data, making important contributions to the fields of AI and public health.

The analysis of different language models provided valuable insights into how these models balance readability, complexity, and accuracy. Each model seems to prioritize different aspects, reflecting distinct approaches to content generation (22). In this context, the current study demonstrated ReKa Core stands out for producing the longest and most detailed responses. As an advanced model, it approaches the frontier models in both automatic and blind human evaluations, emphasizing its potential to enhance diagnostics and disease management. In contrast, models like Llama 3 and Gemini focus on brevity, producing significantly shorter responses, which is consistent with previous literature (23).

Interestingly, a connection between word count and readability emerged. Models like ReKa Core and Bing Chat, which generate longer responses, maintain relatively high FRES values, indicating that their detailed responses are still fairly easy to read (24). On the other hand, models such as Llama 3 and Perplexity, which produce shorter or more moderately long responses, resulted in more complex and less readable text, as reflected by their lower FRES scores (25).

When we consider the FKGL results, this balance becomes even clearer. ScholarGPT and Perplexity generated content at a level suiTable for advanced college readers, making them ideal for academic or technical tasks (21,24,25). In contrast, Llama 3, with a much lower FKGL, produced simpler text appropriate for middle school-level readers. This diversity shows how models are tailored for different audiences. Models like ChatGPT 4.0 and Bing Chat, which produced content at a high school or early college reading level, are particularly versatile, since they are accessible to a broad audience while still being sophisticated enough for more demanding tasks (24).

The analysis of semantic similarity also offers valuable insights into how closely the models' responses align with the original input. Bing Chat performed exceptionally well in this regard, achieving the highest Cosine similarity score. However, its relatively high Levenshtein distance suggests that, although it maintains semantic accuracy, it often rephrases or restructures the input, possibly to enhance readability (26). ReKa Core, while producing highly detailed and readable content, exhibited the greatest lexical divergence, meaning it tends to deviate more from the original text structure, which might be useful in some contexts but could reduce precision in others.

Models like ChatGPT 4.0 and Claude managed to balance semantic coherence and readability effectively. Both models scored well in terms of similarity and readability, making them ideal for tasks where a combination of accuracy and accessibility is important (24,27). This balance allows them to produce content that is not only understandable but also closely aligned with the original source material, which is crucial for tasks that require both clarity and precision (27).

Feedback from specialists further enriches the evaluation of the outputs provided by the LLMs. ReKa Core was rated highly for overall appropriateness, showing its strength in generating comprehensive responses. However, it struggled with strict adherence to WHO guidelines, which points to an important trade-off. While ReKa Core excels in providing in-depth, detailed answers, it may not always align perfectly with more rigid frameworks like the WHO’s (https://doi.org/10.48550/arXiv.2406.06565). On the other hand, GEMINI, which performed poorly in terms of general appropriateness, excelled in adhering to these guidelines, demonstrating that models optimized for specific tasks might compromise in broader applicability (28).

Finally, it is important to note that the ethical issues surrounding the use of LLMs in healthcare are complex and require continuous debate. One of the main ethical principles regarding the use of AI in healthcare includes autonomy, as it ensures the patient's right to make informed decisions about their treatment. Among the ethical challenges, accuracy is crucial to ensure patient safety, avoiding errors that could cause harm, such as unnecessary interventions. Other concerns involve bias, confidentiality and accountability. Improving accuracy, reducing bias and increasing transparency are essential for the responsible use of LLMs, balancing technological advances with patient safety and dignity, promoting trust and good outcomes (29).

This study has some limitations. As a cross-sectional study, the results reflect the performance of the platforms at a specific point in time and may be influenced by future updates to the models, which occur continuously. Although the prompts were standardized, inherent differences between the models might have influenced the generated responses. Furthermore, using only ten questions per platform may not fully capture the variability in the LLMs' performance on this topic. However, despite these limitations, the study offers valuable initial insights into the current performance of LLMs in providing information about syphilis and could serve as a foundation for more comprehensive and complementary future investigations.

## Conclusions

In summary, this analysis highlights the variability in AI model outputs and the importance of selecting the right model for the task at hand. While models like ReKa Core excel in producing detailed and readable content, others like ChatGPT 4.0 and Bing Chat offer a balanced approach that blends readability, accuracy, and semantic alignment. Ultimately, the best choice depends on whether the task prioritizes depth, accessibility, or strict adherence to specific guidelines.

## Figures and Tables

**Table 1:** Questions elaborated using the WHO fact sheets in syphilis as a base.

QUESTIONS
What is syphilis?
How is syphilis transmitted?
What are the symptoms of syphilis?
What are the symptoms of congenital syphilis?
How is syphilis diagnosed?
How is congenital syphilis diagnosed?
How is syphilis treated?
How is congenital syphilis treated?
What are the complications associated with syphilis?
How to prevent syphilis?

**Table 2:** Comparative evaluation of language models in syphilis-related queries in relation to the choice of evaluator, completeness and accuracy of responses.

		ChatGPT 3.5	ChatGPT 4.0	ScholarGPT	GEMINI	Llama 3.0	Bing Chat	Perplexity	Pop-AI	Claude	Reka Core
Choice of evaluator	Model	8	8	7	1	5	6	8	5	5	9
	WHO	2	2	3	9	5	4	2	5	5	1
Completeness LLM answers	Incomplete	2	0	0	4	1	1	0	4	4	0
	Adequate	5	7	8	6	8	7	7	6	5	4
	Comprehensive	1	3	1	0	0	0	3	0	0	5
	No agreement	2	0	1	0	1	2	0	0	1	1
Completeness of WHO answers	Incomplete	3	3	3	3	3	3	2	3	2	4
	Adequate	6	5	7	5	5	7	8	5	7	6
	Comprehensive	0	0	0	0	0	0	0	0	0	0
	No agreement	1	2	0	2	2	0	0	2	1	0
Accuracy of LLM answers	Completely incorrect	0	0	0	0	0	0	0	0	0	0
	More incorrect than correct	0	0	0	0	0	0	0	0	0	0
	Approximately equal, correct and incorrect	0	0	0	0	0	0	0	0	0	0
	More correct than incorrect	3	1	7	7	3	1	1	2	0	0
	Completely correct	6	9	2	3	7	9	9	8	10	10
	No agreement	1	0	1	0	0	0	0	0	0	0
Accuracy of WHO answers	Completely incorrect	0	0	0	0	0	0	0	0	0	0
	More incorrect than correct	0	0	0	0	0	0	0	0	0	0
	Approximately equal, correct and incorrect	0	0	0	0	0	0	0	0	0	0
	More correct than incorrect	5	3	4	4	7	3	3	2	1	2
	Completely correct	5	7	6	6	3	7	7	8	9	8
	No agreement	0	0	0	0	0	0	0	0	0	0

## References

[1] Antal GM, Lukehart SA, Meheus AZ (2002). The endemic treponematoses. Microbes Infect.

[2] Smajs D, Norris SJ, Weinstock GM (2012). Genetic diversity in Treponema pallidum: implications for pathogenesis, evolution, and molecular diagnostics of syphilis and yaws. Infect Genet Evol.

[3] Peeling RW, Mabey D, Kamb ML, Chen XS, Radolf JD, Benzaken AS (2017). Syphilis. Nat Rev Dis Primers.

[4] Yu W, You X, Luo W (2024). Global, regional, and national burden of syphilis, 1990-2021, and predictions by Bayesian age-period-cohort analysis: a systematic analysis for the global burden of disease study 2021. Front Med (Lausanne).

[5] Peeling RW, Mabey D, Chen XS, Garcia PJ (2023). Syphilis. Lancet.

[6] Hook EW (2017). Syphilis. Lancet.

[7] Jankowska L, Adamski Z, Polańska A, Bowszyc-Dmochowska M, Plagens-Rotman K, Merks P (2022). Challenges in the diagnosis of tertiary syphilis: case report with literature review. Int J Environ Res Public Health.

[8] Chow F (2021). Update in: Neurosyphilis. Continuum (Minneap Minn).

[9] Thean L, Moore A, Nourse C (2022). New trends in congenital syphilis: epidemiology, testing in pregnancy, and management. Curr Opin Infect Dis.

[10] Hamill MM, Ghanem KG, Tuddenham S (2024). State-of-the-art review: neurosyphilis. Clin Infect Dis.

[11] Albuquerque G, Fernandes F, Barbalho IM, Barros DM, Morais PS, Morais AH (2023). Computational methods applied to syphilis: where are we, and where are we going?. Front Public Health.

[12] De Souza LL, Fonseca FP, Araujo ALD, Lopes MA, Vargas PA, Khurram AS (2023). Machine learning for detection and classification of oral potentially malignant disorders: a conceptual review. J Oral Pathol Med.

[13] Theodosiou AA, Read RC (2023). Artificial intelligence, machine learning, and deep learning: potential resources for the infection clinician. J Infect.

[14] Soe NN, Yu Z, Latt PM, Lee D, Ong JJ, Ge Z (2024). Evaluation of artificial intelligence-powered screening for sexually transmitted infections-related skin lesions using clinical images and metadata. BMC Med.

[15] Omiye JA, Gui H, Rezaei SJ, Zou J, Daneshjou R (2024). Large Language Models in Medicine: The Potentials and Pitfalls: A Narrative Review. Ann Intern Med.

[16] Heo MS, Kim JE, Hwang JJ, Han SS, Kim JS, Yi WJ (2021). Artificial intelligence in oral and maxillofacial radiology: what is currently possible?. Dentomaxillofac Radiol.

[17] Ossowska A, Kusiak A, Świetlik D (2022). Artificial intelligence in dentistry-narrative review. Int J Environ Res Public Health.

[18] Lee ASD, Cody SL (2020). The stigma of sexually transmitted infections. Nurs Clin North Am.

[19] Babayiğit O, Eroglu ZT, Sen DO, Ucan YF (2023). Potential use of ChatGPT for patient information in periodontology: a descriptive pilot study. Cureus.

[20] Landis JR, Koch GG (1977). The measurement of observer agreement for categorical data. Biometrics.

[21] De Souza LL, Lopes MA, Santos-Silva AR, Vargas PA (2024). The potential of ChatGPT in oral medicine: a new era of patient care?. Oral Surg Oral Med Oral Pathol Oral Radiol.

[22] Kaewboonlert N, Poontananggul J, Pongsuwan N, Bhakdisongkhram G (2025). Factors Associated with the Accuracy of Large Language Models in Basic Medical Science Examinations: Cross-Sectional Study. JMIR Med Educ.

[23] Adams LC, Truhn D, Busch F, Dorfner F, Nawabi J, Makowski MR (2024). Llama 3 challenges proprietary state-of-the-art large language models in radiology board-style examination questions. Radiology.

[24] De Souza LL, Santos-Silva AR, Hagag A, Alzahem A, Vargas PA, Lopes MA (2024). Evaluating AI models in head and neck cancer research: the use of NCI data by ChatGPT 3.5, ChatGPT 4.0, Google Bard, and Bing Chat. Oral Surg Oral Med Oral Pathol Oral Radiol.

[25] Ömür AD, Erdemir İ, Kara F, Shermatov N, Odacioğlu M, İbişoğlu E (2024). Assessing the readability, reliability, and quality of artificial intelligence chatbot responses to the 100 most searched queries about cardiopulmonary resuscitation: An observational study. Medicine (Baltimore).

[26] Tao BK, Hua N, Milkovich J, Micieli JA (2024). ChatGPT-3.5 and Bing Chat in ophthalmology: an updated evaluation of performance, readability, and informative sources. Eye (Lond).

[27] Ermis S, Özal E, Karapapak M, Kumantaş E, Özal SA (2025). Assessing the Responses of Large Language Models (ChatGPT-4, Claude 3, Gemini, and Microsoft Copilot) to Frequently Asked Questions in Retinopathy of Prematurity: A Study on Readability and Appropriateness. J Pediatr Ophthalmol Strabismus.

[28] Quinn M, Milner JD, Schmitt P, Morrissey P, Lemme N, Marcaccio S (2024). Artificial intelligence large language models address anterior cruciate ligament reconstruction: superior clarity and completeness by Gemini compared to ChatGPT-4 in response to American Academy of Orthopedic Surgeons clinical practice guidelines. Arthroscopy.

[29] Pressman SM, Borna S, Gomez-Cabello CA, Haider SA, Haider C, Forte AJ (2024). AI and Ethics: A Systematic Review of the Ethical Considerations of Large Language Model Use in Surgery Research. Healthcare (Basel).

